# State-Dependent Effects of Transcranial Oscillatory Currents on the Motor System during Action Observation

**DOI:** 10.1038/s41598-019-49166-1

**Published:** 2019-09-06

**Authors:** Matteo Feurra, Evgeny Blagovechtchenski, Vadim V. Nikulin, Maria Nazarova, Anna Lebedeva, Daria Pozdeeva, Maria Yurevich, Simone Rossi

**Affiliations:** 1 National Research University, Higher School of Economics, 101000 Moscow, Russia; 2 Department of Medicine, Surgery and Neuroscience, Siena Brain Investigation & Neuromodulation Lab (Si-BIN Lab.), Unit of Neurology and Clinical Neurophysiology and Section of Human Physiology, University of Siena, Siena, 53100 Italy; 3 Department of Neurology, Max Planck Institute for Human Cognitive and Brain Sciences, Leipzig, 04103 Germany; 4 Sainsbury Wellcome Centre for Neural Circuits and Behaviour, University College London, London, WC1E 6BT UK; 5 Centre for Cognition and Decision making, Institute for Cognitive Neuroscience, National Research University, Higher School of Economics, 101000 Moscow, Russia

**Keywords:** Cognitive neuroscience, Motor cortex

## Abstract

We applied transcranial alternating current stimulation (tACS) to the primary motor cortex (M1) at different frequencies during an index–thumb pinch-grip observation task. To estimate changes in the corticospinal output, we used the size of motor evoked potentials (MEPs) obtained by transcranial magnetic stimulation (TMS) of M1 using an online MRI-guided simultaneous TMS-tACS approach. The results of the beta-tACS confirm a non-selective increase in corticospinal excitability in subjects at rest; an increase was observed for both of the tested hand muscles, the first dorsal interosseous (FDI) and the abductor digiti minimi (ADM). However, during action observation of the pinch-grip movement, the increase of corticospinal excitability was only observed for the prime mover FDI muscle and took place during alpha-tACS, while gamma-tACS affected both the FDI and control muscle (ADM) responses. These phenomena likely reflect the hypothesis that the mu and gamma rhythms specifically index the downstream modulation of primary sensorimotor areas by engaging mirror neuron activity. The current neuromodulation approach confirms that tACS can be used to induce neurophysiologically detectable state-dependent enhancement effects, even in complex motor-cognitive tasks.

## Introduction

Compelling evidence indicates that, in the human motor system, the application to the scalp of low-intensity transcranial alternating current stimulation (tACS) with a frequency matching the idling beta rhythm of the resting precentral areas causes an increase in the corticospinal output, measured by the size of the motor evoked potentials (MEPs) elicited by transcranial magnetic stimulation (TMS) of the primary motor cortex (M1)^1–3^. These neurophysiological effects, which point to a less selective motoneuronal recruitment driven by beta activity^1^, have an overt behavioural counterpart as tACS at beta range may slow down some kinematic aspects of voluntary movements^4–7^. On the other hand, gamma-tACS tends to have prokinetic effects on visually-guided motor abilities^5,7^. Taken together, these findings suggest that brain oscillations in the precentral regions are causally, rather than epiphenomenally, linked to motor control.

The effects of tACS are thought to be mediated by entrainment of brain oscillations, with resonance phenomena occurring between the applied frequency of stimulation and the local endogenous rhythms^8^. These local brain rhythms change during the execution of cognitive, motor and perceptual tasks. For example, during motor imagery (MI), a mental process that desynchronizes the beta rhythm of the M1^9,10^, beta-tACS can no longer modulate the corticospinal output, which it can during the resting state^1,2^, when the idling beta rhythm is locally prominent^11,12^. On the other hand, theta- and alpha-tACS increase the corticospinal output (i.e. result in larger MEPs in prime mover hand muscles) during movement imagination, possibly because of the synchronization with theta-mediated working memory (WM) processes necessary to mentally process and “execute” the cognitive task and the synchronization of the attentional alpha rhythm^13^, which promotes cortical changes commonly associated with visual MI^2^. This is in accordance with the idea that tACS effects on the brain are not only frequency-dependent but also state-dependent^14,15^.

In the current study, we address these issues using an action observation (AO) task, which reflects a cognitive ability that engages the motor mirror neuron system (MNS)^16^. Action observation, defined as the perception of the others’ action, produces an activation of the MNS that simulates what would happen if the observer him- or herself were to execute the observed movement (in this case, a pinch-grip action). There is a compelling evidence to suggest that AO produces selective corticospinal facilitation, reflected by an increase in MEP size in the muscles the observer would use (i.e. prime movers) to actually perform the observed action^17–25^. This likely reflects the engagement of facilitatory intracortical circuits^26,27^. Motor imagery and AO partly share a common wide premotor-parietal circuit, whose final pathway is the M1^16,28,29^. In line with a previous combined TMS-tACS protocol adopted during an MI task^2^, we used tACS of the M1 at different frequencies (theta, alpha, beta, gamma and sham) during an AO task, taking into account the state-dependency of tACS effects on brain activity. Our aim was to explore—for the first time in a causal manner––the loco-regional functional relevance of the brain rhythms underpinning the observation of voluntary actions in healthy humans. We also investigated the selectivity of these effects at the cortical level through simultaneous recording of TMS responses during tACS from two hand muscles, the first dorsal interosseous (FDI) and the abductor digiti minimi (ADM), which share peripheral innervation and have a similar cortical representation^30,31^. While the former is fully engaged in the pinch-grip AO examined in this study, the latter is thought not to be involved in this task.

## Methods

All methods were performed in accordance with the relevant guidelines and regulations. Informed consent was obtained from all participants, and the study was approved by the local ethics committee of the Higher School of Economics, Moscow.

### Experimental design

#### Participants

Nineteen right-handed volunteers (8 females,11 males; mean age of 32.27 years) in full health with normal neurological examination results and naive to the purpose of the experiment were included in the study after being screened for suitability for TMS^32^. All of them reported that they had not used drugs or alcohol in the days preceding the experiment. Subjects were asked to sit comfortably in a reclining chair, keeping their arms fully relaxed in a pronate position and their hands resting on the armrests.

#### TMS

TMS was delivered over the left-dominant primary motor cortex (M1) using a MagPro X100 (MagVenture) stimulator with an MCF-B65 induction focal coil (75-mm wing radius) which produced biphasic TMS pulses. The subjects’ individual magnetic resonance imaging (MRI) scans (T1 weighted; 1 mm thickness; sagittal orientation; acquisition matrix 256 × 256) were obtained using a 1.5T MRI scanner (Siemens Magnetom Avanto) and a neuronavigation TMS system (Localite TMS Navigator, Localite GmbH) in an MRI-guided stimulation design which allowed optimisation and recording of the identified TMS brain area (hot spot) and ensured a consistent cortical target throughout the experiment. This system allowed identical positioning of the TMS coil within and across experimental sessions. The coil was held tangentially to the scalp with the handle pointing backwards and laterally angled at 45° from the midline sagittal axis of the participant’s head. Single TMS pulses were delivered in order to find the optimal hotspot for the FDI muscle (i.e. the scalp point eliciting MEPs at threshold level from the contralateral examined hand FDI muscle)^32^. The resting motor threshold (RMT) for the given hot spot was determined as the minimal stimulator output evoking contralateral FDI MEPs of at least 50 µV in a resting muscle with 50% probability^33^. Once the hot spot was determined, it was marked on the scalp with a pencil to facilitate the application of the tACS target electrode.

#### tACS

**Figure 1:**
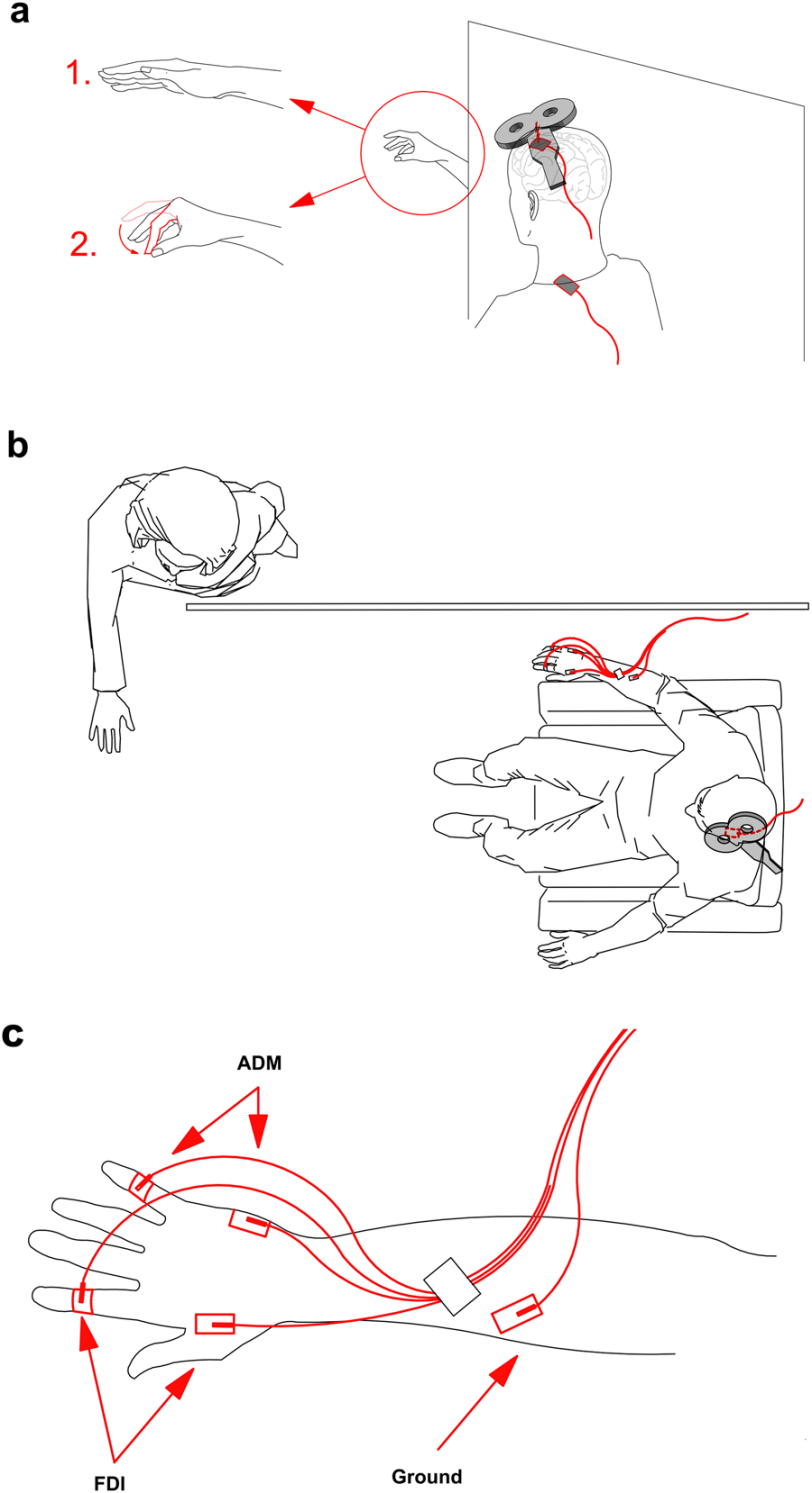
Experimental design. (**a**) Combined TMS-tACS approach: the TMS coil is placed over the target tACS electrode on the M1 area. The reference tACS electrode is placed over the ipsilateral shoulder. The subject’s task is to observe either a steady hand or a pinch-grip action. (**b**) Top view of the design: the actor’s body is hidden by white curtains which are placed to the right of the subject so that the subject observes only the hand movement. (**c**) EMG montage: g–AgCl adhesive electrodes placed on the belly of the right FDI and ADM muscles.

A battery-driven current stimulator (BrainSTIM, EMS Medical, Italy) was used to deliver tACS through surface saline-soaked sponge electrodes (size, 5 × 7 cm). Rubber straps around the head guaranteed stable electrode–scalp contact. As in our previous studies^1,2^, the centre of the target electrode was placed over the left M1 hotspot determined by the TMS procedure. Since it has recently been shown that tACS extracephalic montage allows a clear physiological entrainment of the primary motor cortex activity^34^, we placed the return electrode over the ipsilateral shoulder to avoid any possible side effect from the reference electrode on the near sensorimotor areas (Fig. 1a). Stimulation intensity was adjusted to 1 mA (peak-to-peak) with no DC offset to avoid possible side effects such as a perception of flickering lights, which tACS can induce at a range of 5 to 40 Hz^35,36^. Moreover, as the amplitude of stimulation is the main factor determining the intensity of neurosensory side effects including tingling and itching sensations, in our study, the electrode was rather large (5 × 7 cm), thus current density distribution was ≈14.2 µA/cm^2^. This is in line with a recent study showing that tACS is unlikely to induce neurosensory effects when delivered at lower intensities and far from the retina, i.e. far from prefrontal sites^37^. To minimise skin sensations, the electrodes were continuously soaked with saline solution. Impedances were kept well below 10 kΩ throughout the stimulation sessions. At the end of the experiment, all subjects were asked whether they had perceived any tingling or itching sensation on the skin. None of them reported any of the aforementioned effects. Even during the training, subjects were barely able to perceive whether stimulation was on or off. Moreover, none of them reported perceiving differences between stimulation sessions (frequencies).

#### Combined TMS/tACS setting and procedure

Single pulses of TMS were delivered through the sponge electrode overlying the left M1 during ongoing tACS to index corticospinal excitability^38^ (Fig. 1a). Motor evoked potentials were recorded through Ag–AgCl adhesive electrodes placed on the belly of the right FDI, which is the prime mover for the pinch-grip action of the AO task^18,22^, and the ADM, which shares the cortical representation of the FDI muscle but is not involved in the observed task (Fig. 1c). Recordings started at least 20 s after the beginning of tACS delivery. The TMS intensity was adjusted to 120% of the individual RMT to ensure MEP elicitation even in the combined (TMS-tACS) setup (i.e. without active tACS but still applied over the sponge electrode overlying the left M1)^38^. An average of 15.5 (+/−2.5 SD) artefact-free MEPs per condition, randomly spaced at intervals of at least 5 s, were recorded using BrainAmp amplifiers and BrainVision Recorder software (Brain Products GmbH, Munich, Germany). During the data acquisition, signals were band-pass filtered between 0.016 and 1000 Hz and digitised at a rate of 5 kHz with a dynamic range of ±3.28 mV (with resolution 0.1 µV). Motor evoked potentials were discarded from post-processing if an electromyography (EMG) burst of even minimal amplitude (i.e. 50 uV) preceded the TMS by 300 ms or if there was an MEP-to-MEP onset latency jitter of at least 2 ms. This jitter accompanies any subliminal muscular activation, which can bias the MEP amplitude^39^. Each stimulation session lasted no more than 90 s. The low intensity of tACS ensured that subjects did not feel any scalp sensation and were blind to stimulation frequencies. The experimenters who performed the offline MEP analysis were also blind to the condition of tACS applied. Throughout the experiment, the neuronavigation system was used to allow identical positioning of the TMS coil within and across the experimental conditions. The system detects any difference in spatial coil location and orientation (three rotation angles) with respect to the initial pulse with a tolerance of 2 mm for each dimension. This procedure provides three-dimensional online information on the initial and actual coil placements, while minimising the variability of TMS-induced electric fields directly measured within a scalp model^31,40^.

#### Task

Before beginning the experiment, 10 min of practice familiarised the subjects with the task and the brain stimulation procedures^22^. For the resting task, complete muscular and mental relaxation was requested of every subject. This condition was referred to as “rest.” During the AO task, subjects were asked to remain fully relaxed (i.e. to ensure an absence of EMG activity in the right arm muscles) while observing the experimenter’s right hand (the experimenter presented the action from the visual hemifield contralateral to the stimulated left-dominant M1) performing an index–thumb pinch-to-grip movement (Fig. 1a,b). A right-facing body view of human motion is better represented in the right visual hemifield of the observer^41^. In order to control any external distraction that may have drawn a subject’s attention to the whole body of the experimenter (who was performing the hand movement), apart from the right hand, the body of the experimenter was covered by a white curtain (Fig. 1b). Hence, subjects were able to monitor only the hand movement without paying attention to the whole body of the performer. Before starting the tACS experimental sessions, subjects underwent two randomised control condition sessions to check the stability of non-conditioned MEPs: “rest_hand” and “rest_no hand.” The former required the subjects to observe the unmoving (steady) hand of the experimenter. The latter was a resting session with no visual input for the subject (Fig. 1a). These two conditions served as controls to test whether the subjects’ resting state MEPs were affected by the observation of a steady hand. During the analysis (see below), this part was subsequently refereed as “baseline” for normalisation of the data collected during the tACS experimental part. For the subsequent tACS part of the experiment, 10 conditions were run: for both the AO and rest conditions, tACS was delivered to the left primary motor cortex at 5 Hz (θ band), 10 Hz (α band), 20 Hz (β band), 40 Hz (γ band) and sham (placebo) (counterbalanced conditions/frequencies). For the sham stimulation, we applied sinusoidal low-frequency transcranial random noise stimulation (tRNS) between 0.1 and 100 Hz for 30 s with a 30 s fade out in order to give our subjects a sensation that stimulation was taking place. The reason for this was that low-frequency tRNS includes all the used physiological-like frequencies of our protocol and is still sinusoidal. Moreover, it had already been used in a previous study^42^. Low-frequency tRNS applied for a short duration has been shown not to affect cortical excitability^43,44^. With regard to the tACS, tRNS induces low scalp sensations^45^ and subjects did not realise that stimulation faded out during the experiment. For each experimental session, MEP measurements were simultaneously collected from the FDI and ADM muscles, contralaterally to the stimulated M1. During the tACS, TMS pulses were applied at intervals of 5–8 s to avoid any short-term conditioning effect^2^. Moreover, they were delivered 1–2 s after the initiation of the rest task and during the closure phase of the pinch-grip for the AO task. The experimenter who handled the TMS coil and the experimenter (actor) who performed the movement were the same throughout the entire experiment and data collection process. They underwent intensive training during several pilot sessions before the experiment to coordinate with one another as the timing of the movement had to be roughly the same throughout the protocol. This was continuously monitored via a chronometer by a third experimenter. Such an ecological procedure had already been used to investigate corticospinal excitability changes related to AO^18,21,31,46^. Each TMS pulse was delivered 1–2 s after the initiation of the rest or the AO condition (closure phase of the pinch-grip) following a verbal “go” command^46^.

### Data processing and statistical analysis

A 10-Hz high-pass filter was applied before the extraction and measurement of the MEPs. After the exclusion of MEPs containing artefacts, excessive latency jitter or muscular activity (as described above), the peak-to-peak maximal amplitude of each MEP was calculated offline. The raw amplitude data from the FDI and ADM muscle MEPs were then averaged for each condition. To obtain optimal control over the results of the main experiment, separate one-way repeated measures ANOVAs were performed for the FDI and the ADM data for the two control condition sessions (rest_hand and rest_no hand) to test any resting state differences in subjects due to the observation of a steady hand. Having assessed the absence of any significant effect (see the results section), data from the two control conditions were collapsed and averaged into a new baseline condition. Thus, all the tACS effects on the size of the MEPs recorded during the experimental part (tACS main protocol) were analysed as percentage changes in the mean peak-to-peak amplitude of the collapsed baseline (100%) for both the FDI and ADM muscles^22,46^. In order to avoid any confirmation or hindsight bias resulting from a multiway ANOVA^47^, as a further control, we tested whether the ADM muscle served as a control for the FDI muscle (prime mover for the AO task) during the no-stimulation condition (sham) by running a separate two-way repeated measures ANOVA with the independent factors condition (rest, AO) and muscle (FDI, ADM). Afterwards, we focused on the tACS effects for the FDI and the ADM responses separately by entering the data into two separate two-way repeated measures ANOVAs (i.e. one for each tested muscle), with the independent tACS factors (θ, α, β, γ and sham) and conditions (rest, AO). We also tested whether any of the tACS conditions boosted the MEP increase induced by AO relative to rest. Therefore, the results for rest were subtracted from those of OA for all tACS factors and a one-way ANOVA (θ, α, β, γ and sham) was run on the resulting data for the FDI and the ADM, respectively. The Huynh-Feldt correction was applied where necessary to compensate for the violation of the assumption of sphericity. In the presence of significant interactions and due to the exploratory nature of the study, corrected pairwise comparisons were performed using Fisher’s LSD test to limit the experiment-wise error rate to α and to maximise the power of the test for detecting pairwise differences. The level of significance was set at p = 0.05.

## Results

### Control conditions (no tACS)

The ANOVA comparing the MEP amplitude for the two control conditions (rest_hand and rest_no hand) showed no significant differences for either the FDI muscle [F(1,18) = 0.178, mean square error (MSE) = 5957.439, p = 0.678, partialEta2 = 0.010] or the ADM muscle [F(1,18) = 0.121, MSE = 305.049, p = 0.732, partialEta2 = 0.007]. This suggests that the observation of a steady hand did not modulate motor cortex excitability.

The two-way repeated measures ANOVA, which compared two levels of the condition factor (rest, AO) with the two muscle levels (FDI, ADM), showed no effects for condition [F(1,18) = 2.424, MSE = 14786.625, p = 0.137, partialEta2 = 0.314] or muscle [F(1,18) = 2.273, MSE = 11943.275, p = 0.149, partialEta2 = 0.298]. The interaction effects between the two factors were significant [F(1,18) = 7.179, MSE = 16264.010, p = 0.015, partialEta2 = 0.717]. Post hoc comparisons revealed a near-significant effect (p = 0.054) as an index of the increase in the corticospinal output in the FDI compared to the ADM for the AO condition. A significant effect (p = 0.042) highlighted an increase in the corticospinal output during AO versus rest for the FDI muscle but not for the ADM muscle. This confirmed that our experimental manipulation was effective.

### Main conditions (tACS effects)

**Figure 2:**
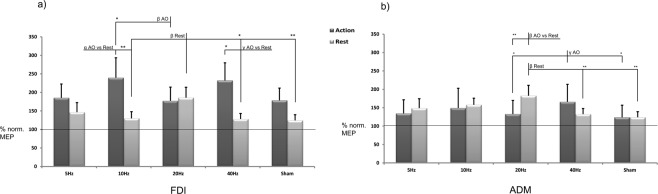
Results. Percentage changes versus baseline MEP amplitude values (raw data) obtained under different experimental conditions. Error bars represent SEM. Asterisks denote significant effects (*p < 0.05, **p < 0.01). Note the frequency- and state-dependent effects of tACS: (**a**) The effects on the FDI muscle: tACS delivered at β range (20 Hz) on the M1 increases the corticospinal output versus most of the other conditions at rest; α-tACS and γ–tACS increases the AO effect. (**b**) The effects on the ADM muscle: tACS delivered at β range (20 Hz) on the M1 still increases the corticospinal output versus sham and γ stimulation at rest, while γ–tACS increases the AO effect versus β and sham stimulation during the AO task.

The two-way repeated measures ANOVA on the normalised FDI data (Fig. 2) contrasted five levels of the factor tACS (θ, α, β, γ and sham) with the two condition levels (rest, AO). The analysis showed a main effect of condition [F(1,18) = 6.045, MSE = 169008.208, p = 0.024, partialEta2 = 0.251]. Post hoc comparisons revealed an expected significant and selective effect of AO versus rest (p = 0.024), confirming that, regardless of the tACS application, AO induced a general increase in corticospinal output. No effect of the tACS factor was observed [F(2.12,38.20) = 1.417, MSE = 13866,787, p = 0.255, partialEta2 = 0.073]. The most interesting effects emerged from the interaction of the two factors F(2.91,52.42) = 3.650, MSE = 31300,649, p = 0.019, partialEta2 = 0.169]. Post hoc comparisons revealed that tACS had significant frequency-dependent and state-dependent effects: α and γ stimulation increased the effects of AO versus rest (p = 0.015 and p = 0.026, respectively) (Fig. 2a). Moreover, the effect of α was significantly stronger than that of β stimulation for the AO condition (p = 0.017). A very robust effect emerged for β stimulation during rest. β stimulation induced an increase in corticospinal output with respect to other stimulation conditions [α (p = 0.005), γ (p = 0.016) and sham (p = 0.009)] and a near-significant effect towards θ (p = 0.058) (Fig. 2a) by confirming that tACS delivered at β range drastically boosts the M1 cortical excitability at rest.

The two-way repeated measures ANOVA on the normalised ADM data contrasted five levels of the factor “tACS” (θ, α, β, γ and sham) with the two condition levels (rest, AO). The analysis showed no main effect of condition [F(1,18) = 0.865, MSE = 5157.405, p = 0.365, partialEta2 = 0.046] or tACS [F(4,72) = 2.277, MSE = 7231,467, p = 0.069, partialEta2 = 0.112]. Significant interaction effects emerged between the two factors tACS and condition F(4,72) = 4.532, MSE = 10476,583, p = 0.003, partialEta2 = 0.201]. Post hoc comparisons showed greater corticospinal excitability during β stimulation at rest than during AO (p = 0.006) and, in the rest condition, greater excitability than γ (p = 0.002) and sham (p = 0.000) stimulation. This confirms the effects of β stimulation on subjects at rest. An interesting effect that emerged for γ stimulation during the AO task was an increase in corticospinal output compared to β (p = 0.050) and sham (p = 0.023) (Fig. 2b) (see Supplementary Information FDI Table S1 and ADM Table S2). This effect is in line with the FDI results which highlight that, in this case, the γ effect is not muscle specific (see discussion).

### AO minus rest (tACS effects)

For the FDI, the one-way ANOVA (θ, α, β, γ and sham) showed a main effect of tACS [F(2.48, 44.70) = 3.65, MSE = 73407,972, p = 0.026, partialEta2 = 0.707]. Post hoc comparisons revealed a difference between β and α (p = 0.006) and between β and γ (p = 0.016). In line with the previous analysis (main conditions, tACS effects), these data confirmed that tACS affects MEPs through β, α and γ stimulation. However, no significant effect emerged for sham although a near-significant effects were observed for β versus sham (p = 0.054) and α versus sham (p = 0.058). For the ADM, the ANOVA showed a main effect of tACS condition [F(4, 72) = 4.53, MSE = 20953,165, p = 0.003, partialEta2 = 0.929]. Post hoc comparisons revealed a difference between β and α (p = 0.015), β and γ (p = 0.002) and β and sham (0.002), confirming the general tACS effect (MEP enhancement) due to β stimulation.

## Discussion

The size of MEPs reflects the magnitude of the output volley evoked by a fixed test stimulus applied to the cortex, which trans-synaptically activates corticospinal neurons^48,49^. In the current study, we used TMS of the M1 to assess the level of corticospinal excitability by measuring changes in MEP size from two hand muscles (FDI, ADM) during rhythmic tACS application at different frequencies and during different states of brain activity (during rest and AO). As expected, and in line with many previous studies^21,22,26,27,50,51^, AO selectively increased the corticospinal output to the FDI muscle that the observer would activate to perform the observed action; this phenomenon did not take place for the ADM muscle, which is not activated during a pinch-grip action.

The effects induced by tACS are complex as a result of i) interactions between external frequency and spatial specificity at the cortical level and ii) state-dependencies due to varying degrees of cognitive engagement of the subjects. When subjects were quiescent (i.e. with their muscles at rest and not engaged in the AO task), the application of tACS in the β range confirmed a general increase in corticospinal output^1–3^ both for the FDI and for the ADM muscle responses. No other tACS frequencies induce any significant modulation of the corticospinal output. Online β–tACS increased the M1 corticospinal excitability of subjects at in both hand muscles (FDI and ADM), which was in line with numerous previous observations^1–3,52^. The possibility that the corticospinal increase during β–tACS could reflect a less selective motoneuronal recruitment^1^ is supported by the fact that beta activity in the motor system is considered to play an antikinetic role as it increases during tonic and postural contractions^53^ but is not functional for the emergence of new upcoming movements in healthy subjects^54^. Moreover, synchronous enhanced beta activity is associated with dysfunctional mechanisms in movement disorders such as Parkinson’s disease^55^. At a mechanistic level, β–tACS may have reinforced emergent network oscillations from reverberating inhibitory loops, which usually oscillate within the beta range^56^. The effects of β–tACS at rest were found to be statistically more robust than those measured during the AO task, which supports previous findings of our group^1,2^. However, the effects of β–tACS are still controversial insofar as they may be dependent on different factors, such as the phase of tACS application, the offline versus online effects of tACS and the idea that the effects of stimulation may be transcutaneous. Increased beta oscillatory activity caused by β–tACS application is supposed to slow down active motor performance^6^. However, this is in contrast with recent evidence showing that β–tACS increased switching transition latency during a bimanual task-set switching paradigm^57^. Additionally, Heise and collaborators (2019) have shown that, as well as inducing online effects, the stimulation had various carry-over effects that must be considered. On the other hand, a previous study investigating the sensorimotor system at rest using our current montage (i.e. TMS over M1) showed that β–tACS increased the corticospinal output in subjects at rest in a phase-dependent fashion, and no carry-over effects were reported^58^. While there are discrepancies between these studies with regard to carry-over effects of tACS, they both show a phase-dependent dependency effect of β–tACS. Therefore, more systematic studies are needed to develop a customised application of tACS to consider phase, brain state, offline versus offline effects and active versus passive tasks. In light of this recent evidence, the transcutaneous effect of β–tACS should also be considered. It has been recently been shown that the effects of tACS may be transcutaneous^59^. Therefore, the neural entrainment may be caused by transcutaneous stimulation of peripheral and cranial nerves. In previous studies, our group has presented solid evidence of the effects of β–tACS at rest^1,2^. Interestingly, while previous studies adopted a bipolar montage (target on M1, Reference CPZ)^1,2^, the current study adopts a monopolar montage (target on M1, reference on the shoulder). Therefore, according to modelling theories, the electric field distribution and target region location should change. If this is true, the shift of electrode position should modulate the effect of β–tACS at rest, which is not the case in the current study in which the effect of beta stimulation remains unchanged. On the one hand, this supports the transcutaneous hypothesis by suggesting that the effects of stimulation may be driven by cranial nerves rather than by specific M1 cortical neurons. On the other hand, it may suggest that, besides the potential transcutaneous effect, having a 5 × 7 cm electrode on the motor cortex may induce a transcranial effect that overcomes the variability of the shifted electrical field. This would mean that part of the M1 cortex underneath the electrode is still stimulated despite the shift in direction of the electrical field. The latter explanation is supported by previous evidence^1^. Indeed, in a study we conducted in 2011, we used a control site to test a potential cortical spread of the current through the brain (right PC active, M1 inactive, reference CPZ, TMS over M1 inactive electrode). We found that, while β–tACS on the M1 induced a robust corticospinal increase, no effect was achieved by stimulating the right PC while recoding TMS-induced MEPs over the M1 electrode (location)^1^. This evidence suggests to us that β–tACS, at least with for the current montage and setup, likely has more transcranial effects than transcutaneous effects and produces a robust replicable effect in subjects at rest.

The current neuromodulatory approach helps to further unveil the functional relevance of brain rhythms in precentral brain regions during AO. The statistical analysis reveals weak and mixed effects of tACS, which certainly require replication in follow-up studies. However, we believe that it is important to provide a speculative interpretation of the data. Given the novelty of our results in relation to the existent literature on transcranial oscillatory stimulation in the motor system, we believe it is necessary to offer a detailed discussion of the mixed effects related to the interactions between the muscles involved, tACS frequencies and subjects’ cognitive engagements.

**Figure 3:**
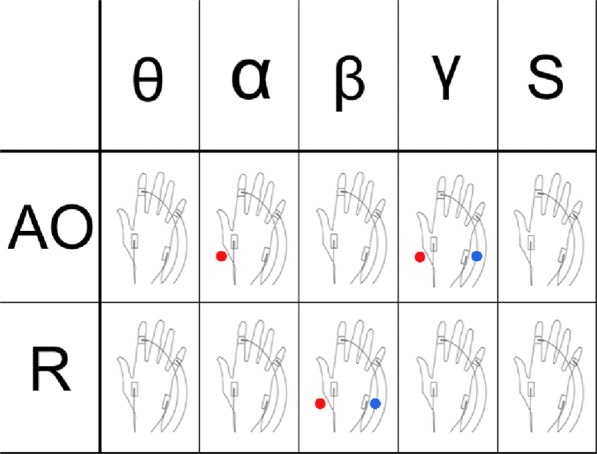
Scheme depicting tACS effects. Red dots represent effects on the FDI muscle while blue dots represent effects on the ADM. Note that the effects of tACS are not only frequency- and state-dependent but also muscle specific.

The analysis of the FDI muscle shows that tACS at α frequency induced a specific increase in the size of MEPs. By contrast, no such increase was found in the analysis of the ADM muscle, although tACS applied at γ frequency induced corticospinal facilitation during AO for both the FDI and the ADM muscles (Fig. 3). It should be noted that, in order to avoid any confirmation or hindsight bias due to the use of a multiway ANOVA^47^, we decided to focus on the FDI and the ADM muscles separately. In addition, since the FDI muscle is the prime mover for the AO used in the current study, the TMS hotspot was defined as the location over which TMS evoked MEPs of highest peak-to-peak amplitude in that muscle (FDI). This location was unique for each subject and was marked according to his or her individual MRI. Therefore, the ADM (no-hotspot) activity may have been affected in terms of stability, thereby introducing variability into the data. Indeed, motor cortical representations of hand muscles vary depending on the angle of the coil used to apply TMS and the muscle to which it is orientated^60^. That is why we first explored whether, during the no-stimulation condition (sham), the ADM muscle served as a control for the FDI (prime mover for the AO task) by running a separate two-way repeated measures ANOVA with the independent factors condition (rest, AO) and muscle (FDI, ADM), which confirmed that the experimental manipulation of the AO versus rest was successful. We then ran two separate ANOVAs for the FDI and ADM respectively. A robust and selective increase in corticospinal output was observed for the FDI muscle durin α-tACS stimulation in the AO condition compared to the rest condition, likely leaving unaltered the descending drive towards the ADM muscle not involved in the motor plan required for the observed pinch-grip action. With regard to MI, AO is usually associated with the suppression of alpha oscillations in the sensorimotor regions^61,62^. However, α-tACS is hypothesised to entrain and thus enhance alpha oscillations^63,64^. These two apparently opposite phenomena can be reconciled by the fact that a strong suppression of alpha oscillations in a given cortical neural pool is associated with an increase of alpha oscillations in neighbouring areas. This is in accordance with the concept of surround inhibition, a relevant mechanism for selectivity of motor control^65^. Moreover, it has been shown that alpha activity is selective to stimuli that may trigger actual movement and increases in oscillatory power as the selective movement demand increases^66,67^. Thus, it can be hypothesised that α-tACS might serve as a further enhancer of spatial selectivity between activation and suppression of motor representations of FDI. Alternatively, few chronometry studies on mu desynchronization and the AO process have shown that mu may desynchronize before^68^, during^69^ or after the observation of an action^70,71^. Additionally, the effects differ depending on whether an action is goal-directed or not^72^. Moreover, recent evidence showed alpha suppression only after 4 s from the onset of the observed grasping movement^73^ and a fast resynchronization of alpha rhythm only after 250 ms of stimulus presentation^74^. Considering our experimental constraints (i.e. using a real ecological hand movement with random timing of the TMS pulse) we can speculate that the increase of MEPs due to single-pulse TMS might have taken place during an event-related desynchronization (ERD) triggered by the previous enhancement of the synchronous alpha rhythm due to tACS. It is likely that a higher event-related synchronization (ERS) will result in a higher ERD^75,76^.

As the outcome of the ANOVA, the corticospinal facilitation in the FDI muscle is superimposed onto the one induced by AO only, a classic finding mediated by mirror neuron activity^21^. This likely reflects the hypothesis that the mu rhythm in the alpha range may specifically index downstream modulation of primary sensorimotor areas by engaging mirror neuron activity. It could represent causal evidence of the neurophysiological underpinning of a system that translates perceptual (“seeing”) representations into action-based representations (“doing”), which is a necessary component of imitation learning^77^.

Like α-tACS, γ–tACS induced an increase in corticospinal motor output measured by the FDI response during the pinch-grip AO, in which the FDI muscle acted as the prime mover. However, at the cortical level, the effect of γ–tACS was less selective than that of α-tACS because the effect extended to the ADM muscle, which is supposed not to be involved in the AO task. No effect was found at rest. Therefore, we infer that this unspecific effect (as far as cortical spatial specificity is concerned) might be related to the general prokinetic effect of gamma activity observed in the basal ganglia-cortical motor loop during voluntary movement^78^. Electrocorticographic findings showed that gamma activity increases in primary motor areas during AO, although to a lesser extent than during action execution. This implies that gamma synchronization across the premotor area and the inferior frontal gyrus would reflect the recruitment of specific neural assemblies in the integration of motor and visual signals^79^ and the binding of different perceptual features into a unique element^80^. Thus, gamma activity seems to be sensitive to the spatial kinematics of human movement and to coordinated versus uncoordinated movement^81^, as well as to movement initiation and movement performance as recently shown by tACS studies^5,7^.

The absence of θ–tACS enhancement on the corticospinal output, demonstrated in a previous study in which subjects were asked to imagine movements (visual MI)^2^, might reflect the absence of WM load during the AO task. In fact, theta activity underlies the organization of sequentially ordered WM items^82^ as in the case of a visual MI task^83^. It is notable that the emerging theta activity during observation of a robot with respect to a human arm action confirms the role of theta in WM processing of novel information^84^.

The differential effects of tACS on motor areas during two different cognitive/motor task, such as MI^2^ and AO, indicates that the entrainment of local brain rhythms is somewhat state-dependent even within overlapping circuitries, as the two tasks share the engagement of many cortical structures and networks, including the ventral premotor cortex, supplementary motor area, inferior and superior parietal lobe and the M1 as the final common pathway^16,50,85^. Here, the different responses of FDI and ADM muscles to alpha- and gamma-tACS may indicate a finer tuning of the state-dependency effect, as the cortical representation of these two muscles may overlap, at least when based on the spatial resolution of TMS mapping^31^. A very selective corticospinal recruitment during AO of movements involving the prime mover muscle for the observed action (FDI) but not the ADM muscle has been documented in previous studies^18,21,22,39^.

The current study makes an important contribution to research on state-dependency. Whereas non-invasive brain stimulation (NIBS), such as TMS, has traditionally been used to test cortical excitability and, in a more advanced fashion, to modulate brain behaviour and physiological signatures in motor, perceptual and cognitive processing (e.g. decision making, WM, long-term memory and language), more recent research on manipulation of the underlying neural activity during different tasks and preconditioning and priming protocols may provide important clues to the possible outcomes (e.g. enhancement and inhibition) that NIBS may induce, specifically the so-called “state-dependent effects”^15,86,87^. Here, we provide further evidence that tACS may also be used to investigate the state-dependent effects of brain electrical stimulation, specifically of the sensorimotor system. By manipulating the motor task (e.g. resting and AO) we have been able to show that different stimulation frequencies influence M1 excitability depending on the state of the subject. In addition, the use of the combined online TMS-tACS approach shows that different motor processing tasks, such as MI and AO, may either share similar responses to a specific frequency, such as 10 Hz stimulation, or show a different frequency-dependent response to either 5 Hz during a MI task^2^ or, as in the current study, 40 Hz stimulation during an AO task. The current evidence together with future investigations may help to disentangle the complexity of the sensorimotor system during rest, MI, AO and real movement. Considered together, these findings highlight the importance of the frequency used to electrically stimulate the cortex to induce physiological and behavioural changes in brain activity. The current study represents a further step in revealing the state-dependent mechanisms of tACS of the sensorimotor system. The neural underpinnings of the mirror neuron effect (triggered by AO) and MI processes have generated significant debate. It is still not clear if MI and AO entail two fully distinct mechanisms or if, and by how much, they overlap^88^ or interact with each other^89^. The current study does not directly compare MI and AO. Since the nature of this study is exploratory rather than hypothesis-driven some limitations need to be taken into account: i) the lack of direct comparisons between AO versus another motor task (e.g. MI), the high number of experimental conditions in the framework of statistical analysis (e.g. excessive number of conditions including control ones, excessive number of factors), ii) the relatively low number of trials per condition (MEPs), iii) the lack of direct comparison between the FDI and ADM muscles, and iiii) the possibility that stimulation effects may be transcutaneous rather than transcranial. Such limitations need further investigation which is crucial to understand reliability of tACS application.

Nevertheless, the current online TMS-tACS approach suggests frequency and state-dependent effects which appear to be task specific, as shown by α modulation of the FDI and γ modulation of both FDI and ADM during the AO task only (Fig. 3). In a previous study, we have shown how α- and θ–tACS modulate MI processing^2^. Whereas α-tACS seems to have an impact on both MI and AO, the results of γ and θ stimulation in the previous and current studies^2^ suggest that these two mechanisms are distinctive. Finally, β–tACS confirmed the corticospinal increase at rest. This study sheds light on the neural underpinning of the sensorimotor system from rest to action. Its findings may be of relevance for neurorehabilitation purposes, where a combined approach of AO and MI has been hypothesisedzed to induce beneficial effects^90^.

## Supplementary information

Supplementary Information

## Data Availability

The datasets generated and analysed during the current study are available from the corresponding author on reasonable request.
