# Author Correction: State-Dependent Effects of Transcranial Oscillatory Currents on the Motor System during Action Observation

**DOI:** 10.1038/s41598-019-54378-6

**Published:** 2019-11-27

**Authors:** Matteo Feurra, Evgeny Blagovechtchenski, Vadim V. Nikulin, Maria Nazarova, Anna Lebedeva, Daria Pozdeeva, Maria Yurevich, Simone Rossi

**Affiliations:** 1 National Research University, Higher School of Economics, 101000 Moscow, Russia; 2 Department of Medicine, Surgery and Neuroscience, Siena Brain Investigation & Neuromodulation Lab (Si-BIN Lab.), Unit of Neurology and Clinical Neurophysiology and Section of Human Physiology, University of Siena, Siena, 53100 Italy; 3 Department of Neurology, Max Planck Institute for Human Cognitive and Brain Sciences, Leipzig, 04103 Germany; 4 Sainsbury Wellcome Centre for Neural Circuits and Behaviour, University College London, London, WC1E 6BT UK; 5 Centre for Cognition and Decision making, Institute for Cognitive Neuroscience, National Research University, Higher School of Economics, 101000 Moscow, Russia

Correction to: *Scientific Reports* 10.1038/s41598-019-49166-1, published online 06 September 2019

The original version of this Article contained a typographical error in the spelling of the author Evgeny Blagovechtchenski, which was incorrectly given as Evgeny Blagoveshchensky. This has now been corrected in the PDF and HTML versions of the Article, and in the accompanying Supplementary Information file.

