# Supplementary material for: State-Dependent Effects of Transcranial Oscillatory Currents on the Motor System during Action Observation
Source: Sci Rep. 2019 Sep 6;9:12858. doi: 10.1038/s41598-019-49166-1 (PMC6731229; doi:10.1038/s41598-019-49166-1)
Supplement: Supplementary file 1 — Supplementary Information [file 41598_2019_49166_MOESM1_ESM.pdf]

State-Dependent Effects of Transcranial Oscillatory Currents on the Motor System during Action Observation

[1,5]Matteo Feurra, [5]Evgeny Blagovechtchenski, [3,5]Vadim V. Nikulin, [1,5]Maria Nazarova, [4]Anna Lebedeva, [1]Daria Pozdeeva, [1]Maria Yurevich, [2]Simone Rossi.

1. School of Psychology, Higher School of Economics, Russian Federation, 10100.

2. Department of Medicine, Surgery and Neuroscience, Siena Brain Investigation & Neuromodulation Lab (Si-BIN Lab.), Unit of Neurology and Clinical Neurophysiology and Section of Human Physiology, University of Siena, Italy, 53100.

3. Department of Neurology, Max Planck Institute for Human Cognitive and Brain Sciences, Leipzig, Germany, 04103.

4. Sainsbury Wellcome Centre for Neural Circuits and Behaviour, University College London, UK, WC1E 6BT.

5. Centre for Cognition and Decision making, Institute for Cognitive Neuroscience, National Research University Higher School of Economics, Russian Federation.

Corresponding author: Matteo Feurra (mfeurra@hse.ru; matfeu@gmail.com), Faculty of Psychology, National Research University, Higher School of Economics. 101000, Moscow, Armyanskiy per. 4, c2 - Room 404. Tel. +79104688329.

SUPPLEMENTARY INFORMATION

Table S1

Tests of Within-Subjects Effects

FDI		Type III Sum of Squares	df	Mean Square	F	Sig.	Partial Eta Squared
Condition	Sphericity Assumed	169008,209	1	169008,209	6,045	,024	,251
	Greenhouse-Geisser	169008,209	1,000	169008,209	6,045	,024	,251
	Huynh-Feldt	169008,209	1,000	169008,209	6,045	,024	,251
	Lower-bound	169008,209	1,000	169008,209	6,045	,024	,251
Error(Condition)	Sphericity Assumed	503252,139	18	27958,452			
	Greenhouse-Geisser	503252,139	18,000	27958,452			
	Huynh-Feldt	503252,139	18,000	27958,452			
	Lower-bound	503252,139	18,000	27958,452			
tACS	Sphericity Assumed	29431,863	4	7357,966	1,417	,237	,073
	Greenhouse-Geisser	29431,863	1,903	15462,793	1,417	,256	,073
	Huynh-Feldt	29431,863	2,122	13866,787	1,417	,255	,073
	Lower-bound	29431,863	1,000	29431,863	1,417	,249	,073
Error(tACS)	Sphericity Assumed	374001,411	72	5194,464			
	Greenhouse-Geisser	374001,411	34,261	10916,185			
	Huynh-Feldt	374001,411	38,204	9789,462			
	Lower-bound	374001,411	18,000	20777,856			
Condition * tACS	Sphericity Assumed	91163,711	4	22790,928	3,650	,009	,169
	Greenhouse-Geisser	91163,711	2,484	36703,986	3,650	,026	,169
	Huynh-Feldt	91163,711	2,913	31300,649	3,650	,019	,169
	Lower-bound	91163,711	1,000	91163,711	3,650	,072	,169
Error(Condition*tACS)	Sphericity Assumed	449562,858	72	6243,929			
	Greenhouse-Geisser	449562,858	44,708	10055,627			
	Huynh-Feldt	449562,858	52,425	8575,299			
	Lower-bound	449562,858	18,000	24975,714			

Table S1. Within-subjects effects (pairwise comparisons) for the FDI muscle.

Table S2

Tests of Within-Subjects Effects

ADM		Type III Sum of Squares	df	Mean Square	F	Sig.	Partial Eta Squared
Condition	Sphericity Assumed	5157,405	1	5157,405	,865	,365	,046
	Greenhouse-Geisser	5157,405	1,000	5157,405	,865	,365	,046
	Huynh-Feldt	5157,405	1,000	5157,405	,865	,365	,046
	Lower-bound	5157,405	1,000	5157,405	,865	,365	,046
Error(Condition)	Sphericity Assumed	107369,552	18	5964,975			
	Greenhouse-Geisser	107369,552	18,000	5964,975			
	Huynh-Feldt	107369,552	18,000	5964,975			
	Lower-bound	107369,552	18,000	5964,975			
tACS	Sphericity Assumed	28925,867	4	7231,467	2,277	,069	,112
	Greenhouse-Geisser	28925,867	2,991	9671,583	2,277	,090	,112
	Huynh-Feldt	28925,867	3,653	7918,847	2,277	,076	,112
	Lower-bound	28925,867	1,000	28925,867	2,277	,149	,112
Error(tACS)	Sphericity Assumed	228701,492	72	3176,410			
	Greenhouse-Geisser	228701,492	53,835	4248,227			
	Huynh-Feldt	228701,492	65,750	3478,340			
	Lower-bound	228701,492	18,000	12705,638			
Condition * tACS	Sphericity Assumed	41906,332	4	10476,583	4,532	,003	,201
	Greenhouse-Geisser	41906,332	3,338	12553,152	4,532	,005	,201
	Huynh-Feldt	41906,332	4,000	10476,583	4,532	,003	,201
	Lower-bound	41906,332	1,000	41906,332	4,532	,047	,201
Error(Condition*tACS)	Sphericity Assumed	166432,146	72	2311,558			
	Greenhouse-Geisser	166432,146	60,090	2769,733			
	Huynh-Feldt	166432,146	72,000	2311,558			
	Lower-bound	166432,146	18,000	9246,230			

Table S2. Within-subjects effects (pairwise comparisons) for the ADM muscle.
